# A novel of WS_2_–MoCuO_3_ supported with graphene quantum dot as counter electrode for dye-sensitized solar cells application

**DOI:** 10.1038/s41598-023-34637-3

**Published:** 2023-05-12

**Authors:** Yonrapach Areerob, Won-Chun Oh, Chaowalit Hamontree, Theeranuch Nachaithong, Supinya Nijpanich, Kongsak Pattarith

**Affiliations:** 1 Department of Industrial Engineering, School of Engineering, King Mongkut’s Institute of Technology Ladkrabang, Bangkok, 10520 Thailand; 2 Department of Advanced Materials Science and Engineering, Hanseo University, Seosan-Si, Chungcheongnam-Do 31962 South Korea; 3 Anhui International Joint Research Center for Nano Carbon-Based Materials and Environmental Health, College of Materials Science and Engineering, Anhui University of Science & Technology, Huainan, 232001 People’s Republic of China; 4 Department of Physics, Faculty of Science, Khon Kaen University, Khon Kaen, 40002 Thailand; 5 Synchrotron Light Research Institute (Public Organization), 111 University Avenue, Muang District, Nakhon Ratchasima, 30000 Thailand; 6 Department of Chemistry, Faculty of Science, Buriram Rajabhat University, Buriram, 31000 Thailand

**Keywords:** Materials for energy and catalysis, Energy harvesting

## Abstract

A novel tungsten disulfide-molybdenum copper oxide composite supported with graphene quantum dots (WM@GQDs) has been synthesized as a counter electrode (CE) for dye-sensitized solar cells (DSSCs) using a simple and low-cost ultrasonication method. The unique structure of WM@GQDs exhibits excellent power conversion efficiency due to its high catalytic activity and charge transport properties. In addition, the graphene quantum dots (GQDs) provide more active sites in the zero-dimensional materials for an I/I_3_^−^ redox reaction which can improve the electrical and optical properties of the composite. The results indicate that the amount of GQDs in the composite affect the effectiveness of solar devices. When 0.9%wt of GQDs was used, the WM@GQDs composite achieved an efficiency of 10.38%, which is higher than that of the expensive platinum CE under the same conditions. The mechanism behind the improved power conversion efficiency (PCE) of the composite sample is also discussed in detail. Therefore, WM@GQDs can be an efficient material to replace platinum in DSSCs as a CE.

## Introduction

The looming shortage of fossil fuels due to annual population growth and the impact of sustained economic growth has made the global population aware of the importance of renewable energy. Among the various renewable energy sources, solar energy is a natural and sustainable energy source that can be used indefinitely. In general, silicon-based solar cell technology will continue to dominate the global market, but researchers are seeking alternative solutions to meet the energy needs of the industrial sector and reduce production costs so that the world's population can access solar energy^1^. One such solution is dye-sensitized solar cells (DSSCs), which are third-generation cells due to their low-cost, simple, and environmentally friendly manufacturing methods^2,3^. DSSCs consist of a photoanode (a semiconductor with a high specific surface area for adsorbed dye), a counter electrode (usually in the form of FTO with platinum), and an electrolyte in the interelectrode space (made of an organic solvent with a redox mediator)^4^. In general, the working principle of DSSCs is that dye molecules, after absorbing a photon, are excited from the ground state to the excited state. Then, an electron is injected into the conduction band (CB) of the semiconductor and transported through an external circuit to the counter electrode. The oxidized dye is regenerated by a redox mediator, and the donated electron from the working electrode reduces the redox species. The cycle is then closed and repeated until illumination occurs^5^.

As mentioned above, the counter electrode (CE) is essential for the proper functioning of DSSCs. Platinum (Pt) is the most widely used CE in DSSCs due to its excellent electrocatalytic performance and high conductivity, which leads to high efficiency in photoelectric transformation^6^. However, Pt is a precious metal, and its stability is insufficient, which can cause it to react with the electrolyte over time^7^. Additionally, it's high-cost limits large-scale production of DSSCs for use in household and industrial sectors. Therefore, researchers are urgently seeking alternative materials to replace Pt. Different kinds of materials, such as carbon-based materials^8^, organic polymers^9^, transition metal dichalcogenides^10^, oxide-based materials^11^, and sulfide-based materials^12^, have been investigated and have shown excellent power conversion due to their superior electrochemical properties.

Graphene quantum dots (GQDs) are promising carbon-based quantum dot materials that are primarily composed of sp^2^ hybridized atoms of nanometer-sized graphene sheets, giving them zero-dimensional properties^13^. GQDs have several desirable properties, including chemical inertness, biocompatibility, stable photoluminescence properties, low resistance, and good redox reversibility. In 2013, Chen et al.^14^ synthesized GQDs-doped polypyrrole (PPy) as the counter electrode. The GQDs-doped PPy film has a highly porous structure and displays higher catalytic current density and lower charge transfer resistance than PPy alone toward the I_3_^−^/I^−^ redox reaction. The DSSC with PPy doped with 10% GQDs showed the highest power conversion efficiency (5.27%), which is higher than that of a Pt counter electrode-based DSSC.

Recently, 2D layered transition metal dichalcogenides (TMDs) such as MoS_2_, TiS_2_, WS_2_, and MoSe_2_ have been considered as potential counter electrodes (CEs) in DSSCs due to their low cost, environmentally friendly production, simple process, high electrocatalytic activity, and chemical stability^15,16^. Among these, WS_2_ has received particular attention due to its thin nanosheets with a large surface area, which can increase the number of active sites on the surface and improve its activity in solar cell applications. In 2018, Yuan et al.^17^ prepared MoSe_2_ nanoflowers via a facile and economical hydrothermal method as CEs in DSSCs. They reported that the DSSCs assembled with MoSe_2_ CEs achieved high power conversion efficiency (PCE = 7.01%) for I_3_^−^ reduction compared to standard Pt due to their abundant active sites and stable chemical properties. However, various methods have been used to increase the electrical conductivity of TMDs, such as doping with polymers and creating composites with carbon-based and other compounds. In 2021, Silambarasan et al.^18^ fabricated MoS_2_ and N-doped graphene quantum dots anchored on reduced graphene oxide (rGO) through a two-step hydrothermal method for use as counter electrodes in DSSCs. The N-GQD@MoS_2_@rGO showed a photovoltaic power conversion efficiency (η) of 4.65%. This was due to the superior catalytic property of the material, which was attributed to the increased electrochemical active sites and electrical conductivity of the rGO and MoS_2_. In addition, 2D carbon materials act as a platform to reduce the agglomeration of MoS_2_ and enhance the electrochemically active sites.

In this work, we synthesized a novel WS_2_-MoCuO_3_ composite supported with graphene quantum dots (WM@GQDs) through a hydrothermal method for use as a counter electrode (CE) in dye-sensitized solar cells (DSSCs). These nanocomposites were characterized in terms of their morphological and electrochemical properties using techniques such as XPS, Raman, XRD, FESEM, HRTEM analysis, and EIS. We optimized the percent weight of GQDs in the WM@GQDs composite and investigated the resulting power conversion efficiency (PCE). The 1.5% WM@GQDs composite achieved a PCE of 10.00%, which is comparable to that of the Pt standard (7.00%) due to the presence of GQDs in the composite material, which increases the rate of charge transfer processes through the electroactive layer in the WM@GQDs composite. We propose that the WM@GQDs composite could be a promising alternative CE material for DSSCs.

## Experimental

### Chemicals and materials

Graphite powder (325 mesh) was purchased from Sigma-Aldrich. All chemicals used in this work were commercially available: Ammonium persulfate ([NH_4_]_2_S_2_O_8_), potassium bromide (KBr, ≥ 99%), and acetonitrile (C_2_H_3_N, 98%, Sigma-Aldrich) were used as the reagents. Chloroplatinic acid hexahydrate (H_2_PtCl_6_ × 6H_2_O) and sodium borohydride (NaBH_4_) were purchased from Sigma-Aldrich. All chemicals were used without further purification (purification of all the chemicals is 99.0%).

### Prepare the WS_2_/MoCuO_3_@Graphene quantum dot CE (WM@GQDs)

Graphene oxide was synthesized by Hummer’s method. Graphite powder (10 g) was added to the 45 mL of concentrated sulfuric acid (H_2_SO_4_) under continuous magnetic stirring in the ice bath. After mixing for 15 min, 2.25 g KMnO_4_ was added to the solution. Then, heat the mixture solution to 50–60 °C while stirring for 2–3 h. The mixture was placed in the ultrasonic bath for 30 min at room temperature and slowly add hydrogen peroxide (H_2_O_2_) while stirring. This step produces a strongly exothermic reaction and causes the mixture to bubble vigorously. After 2 h of stirring, the mixture is washed with deionized water to remove excess acid, nitrate, and sulfate ions. The resulting graphite oxide is dried under a vacuum to obtain a powdery substance.

The typical preparation process for graphene quantum dots (GQDs) is as follows: 500 mg of graphene oxide (GO) and 100 µL of dimethylformamide (DMF) were dissolved in 10.0 mL of deionized water under conditions of vigorous stirring. The mixture was then mixed with 3 mL of ammonia and sonicated for 20 min. Afterward, the mixture was transferred into a Teflon-lined autoclave and heated at 140 °C for 48 h^19^.

MoCuO_3_ nanowires were synthesized through a hydrothermal method. 1.5 g of ammonium molybdate and 1.0 g of copper (II) oxide were dissolved in deionized (DI) water, and 5 mL of HNO_3_ was added to the solution. After 10 min of stirring, the solution was transferred to a 100 mL Teflon-lined stainless steel autoclave, sealed, and placed in an air-drying oven heated at 180 °C for 12 h. The final product was rinsed with DI water until the waste solution was neutral, and the precipitate was vacuum dried.

**Figure 1:**
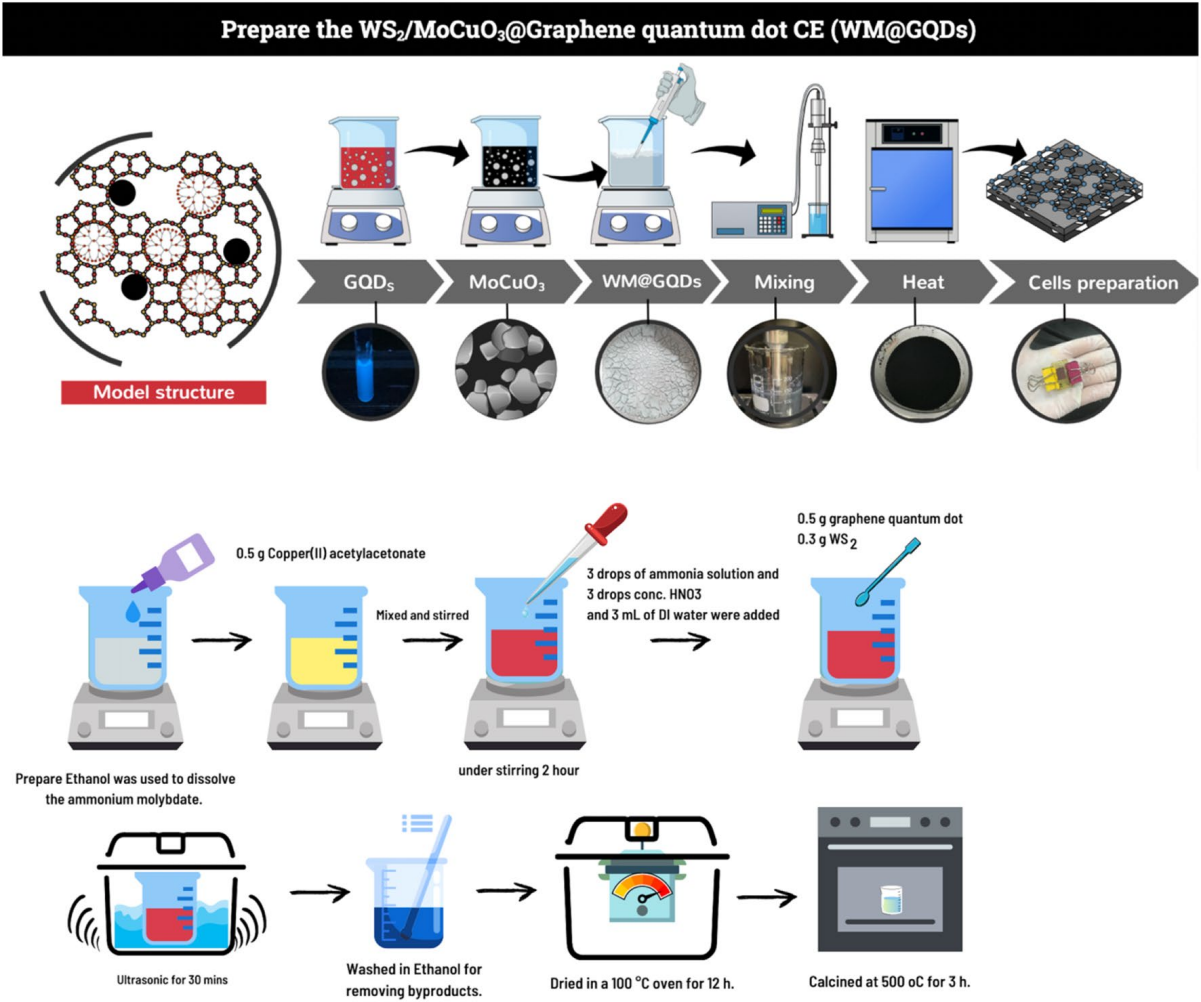
Schematic diagram representing the method of preparation of the WS_2_/MoCuO_3_@graphene quantum dot CE (WM@GQDs).

The WS_2_ powder, MoCuO_3_, and GQDs were then dispersed in DI water, stirred for 30 min, and ultrasonicated for 1 h. The uniform mixture was then transferred to a 100 mL Teflon-lined stainless-steel autoclave, centrifuged at 4000 rpm, washed with DI acetone, and dried for further use. Four concentrations of GQDs in WM@GQDs, i.e., 0.3 wt% GQDs, 0.6 wt% GQDs, and 0.9 wt% GQDs, were prepared using the same procedure. Figure 1 illustrates the method of synthesis of WM@GQDs nanocomposites.

### Fabrication of solar cell device

Typically, a fluorine-doped tin oxide (FTO) glass substrate is used to design the sandwich-type solar cell device, and the doctor blade method is used to deposit both the photo and counter electrodes^20^. First, 0.5 g of WM@GQDs is dispersed in 5 mL of ethanol and ground well for 30 min using a mortar and pestle. The slurry for each counter electrode is coated on the FTO substrate and then dried at 80 °C before being annealed at 400 °C for 2 h. The same fabrication method is followed to prepare all other photoanodes (TiO_2_, commercial P25). The N719 dye solution is immersed in the TiO_2_ PE electrodes for 24 h and annealed at 400 °C for 2 h. Finally, the electrolyte solution (I^−^/I_3_^−^) is dropped through one of the holes between the two electrodes, which are then sealed with silicon sticks to prevent evaporation.

### Characterization techniques

The crystalline properties of the synthesized nanocomposites were studied using an X-ray diffractometer (SmartLab, Rigaku) under Cu Kα radiation. The morphology of the synthesized nanocomposite was investigated using field emission scanning electron microscopy (FESEM: Thermo Fisher Scientific, Apero2) and transmission electron microscopy (TEM, JEOL JEM-2100). The surface elemental composition and oxidation states were analyzed using X-ray photoelectron spectroscopy (XPS; PHI5000 VersaProbe II ULVAC-PHI, Japan) at the Synchrotron Light Research Institute (SLRI) in Thailand. Raman spectra were recorded using a microscopy Raman spectrometer (NRS-7100, JASCO). The current–voltage (I–V) characteristics of the fabricated photovoltaic devices were studied using a Class A solar simulator workstation (SL-50A-WS, SCIENCE TECH) at AM1.5 (sunlight of 100 mW/cm^2^). For electrochemical impedance spectroscopy (EIS) measurements, the electrolyte solution consisted of 0.05 M I_2_ and 0.5 M LiI in acetonitrile. Nyquist plots were recorded over a frequency range of 0.1 Hz–100 kHz and measured at 250 mV to negate the limited regime of mass transport.

## Results and discussion

### Characterization analysis of WM@GQDs

#### XRD analysis

**Figure 2:**
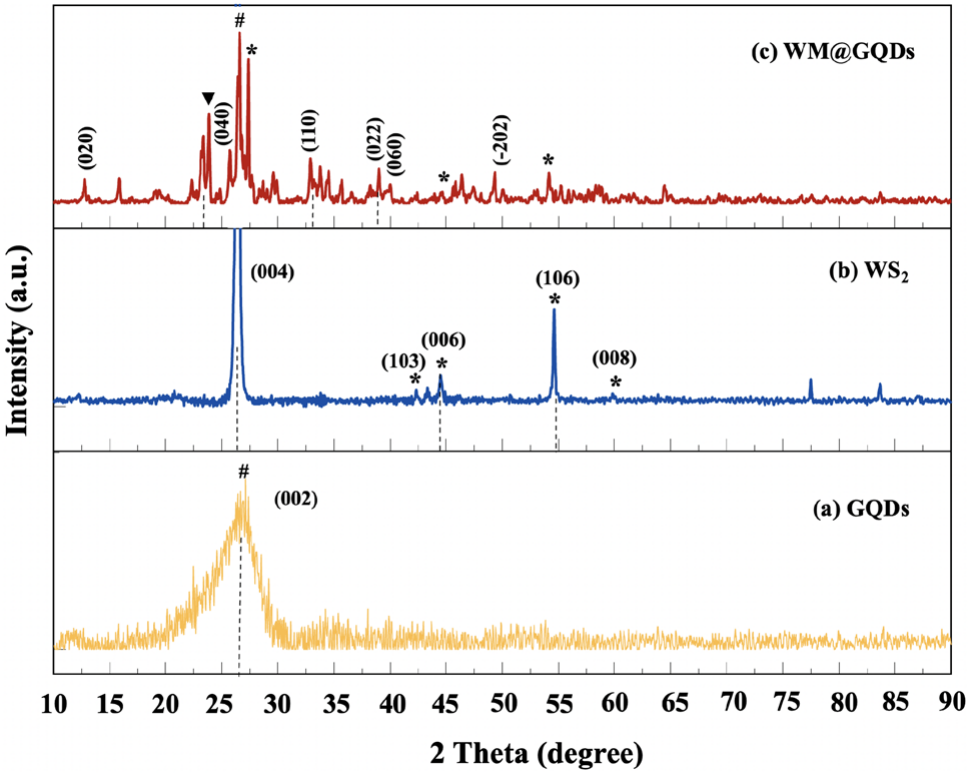
XRD patterns recorded for (**a**) GQDs, (**b**) WS_2_, and (**c**) WM@GQDs.

The crystalline properties of the synthesized GQDs, WS_2_, and WM@GQDs were studied using the XRD analysis technique, as shown in Fig. 2. Analysis of the pattern recorded for GQDs reveals an amorphous structure, which is evident from the diffraction peak at 2θ = 25.8°, that corresponds to the (002) plane of the sp^2^-hybridized carbon atoms in GQDs^21^. In addition, the diffraction peaks at 25.7°, 42.9°, 44.7°, and 54.9° corresponded to the (004), (103), (006), (106), and (008) planes, respectively, which were indexed as the hexagonal phases of WS_2_ (JCPDS FILE: 87–2417 and 84–1398)^22^. The patterns corresponding to WM@GQDs show peaks at 12.5°, 32.5°, 38.3°, and 50.1°, corresponding to the (020), (110), (022), and (-202) planes, respectively. XRD analysis confirmed that the WM@GQDs composites were formed.

#### Surface morphology analysis

**Figure 3:**
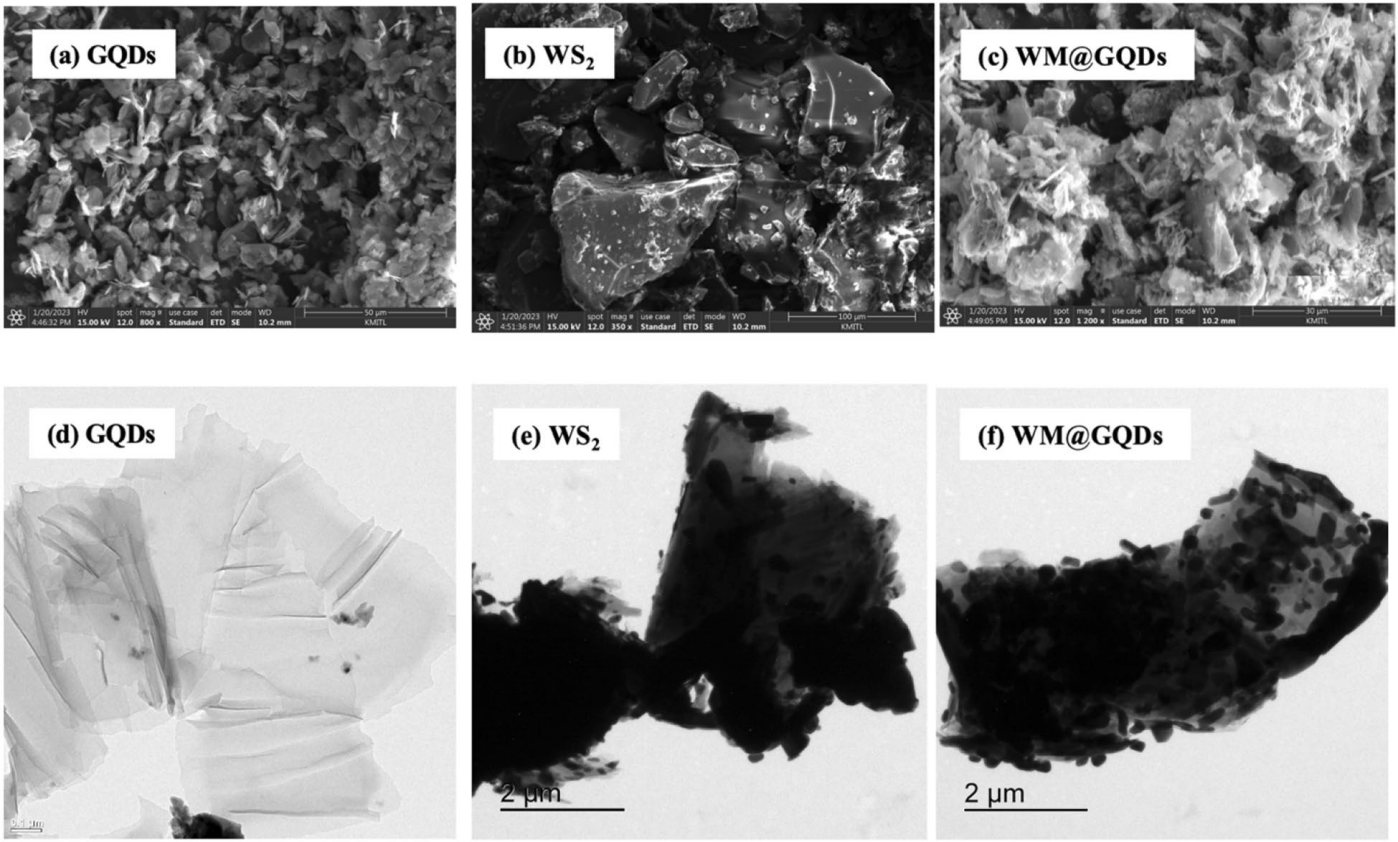
SEM and TEM image of (**a**,**d**) GQDs, (**b**,**e**) WS_2_, and (**c**,**f**) WM@GQDs.

The surface morphology of GQDs, WS_2_, and WM@GQDs was investigated using the FESEM imaging technique, as shown in Fig. 3a–c. Figure 3a shows a monodispersed and uniform spherical structure of the prepared GQDs, with an average size of approximately 10 nm. Moreover, Fig. 3b clearly shows the presence of flake-like WS_2_ nanosheets on the surface. The size of WM@GQDs ranged from 2 to 10 nm for the WS_2_-MoCuO_3_ system embedded with GQDs. The microstructure of WS_2_ was investigated using the high-resolution TEM technique, and the results are presented in Fig. 3d–f. Figure 3d presents the HRTEM image of the GQDs, which clearly indicates the formation of stacked nanosheets. The WS_2_ nanosheets were also observed to agglomerate into larger clusters, as shown in Fig. 3e. The high-resolution TEM images confirm the formation of ultrathin sheets of WS_2_, GQDs, and MoCuO_3_ embedded in the composite.

#### FTIR analysis and Raman spectra

**Figure 4:**
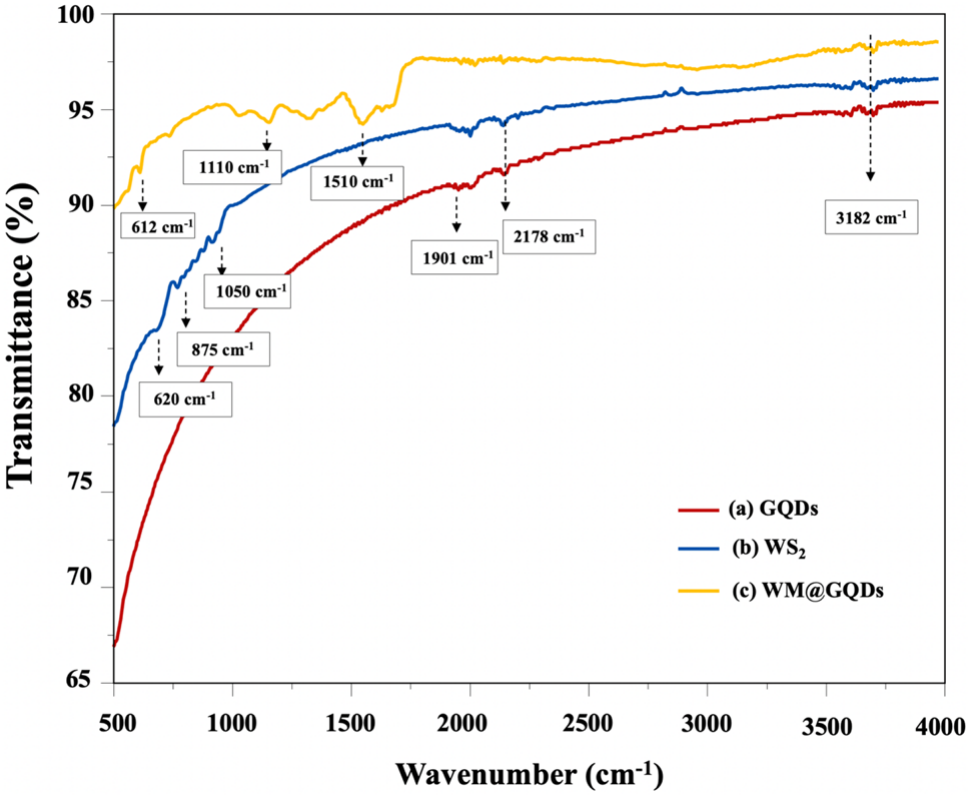
FTIR profiles recorded for (**a**) GQDs, (**b**) WS_2_, and (**c**) WM@GQDs.

FTIR and Raman spectroscopy were used to characterize the structure and composition of the composite. Figure 4a presented the FTIR spectral profiles recorded for GQDs, WS_2_, and WM@GQDs. The profiles recorded for GQDs present bands that confirm the graphene-like structure of the materials (the C=C backbone vibrations appear at 1921 cm^−1^, and the presence of the oxygen-containing groups (such as O–H) was represented by the stretching vibrations at 3182 cm^−1^. The C=O stretching vibrations appeared at 2178 cm^−1^. The profiles recorded for pure WS_2_ exhibits three characteristic absorption peaks at 620 cm^−1^ and in the region spanning 875–1051 cm^−1^. These peaks were attributed to the W–S and S–S bonds in WS_2_^23,24^. In addition, the spectral profiles recorded for MoCuO_3_ revealed the presence of Mo–O stretching vibrations at approximately 1110 cm^−1^. The band at approximately 612 cm^−1^ was associated with the vibrations of the O atoms in the MoCuO_3_ lattice. Signals characteristic of both components were clearly observed in the spectral profiles recorded for WM@GQD.

**Figure 5:**
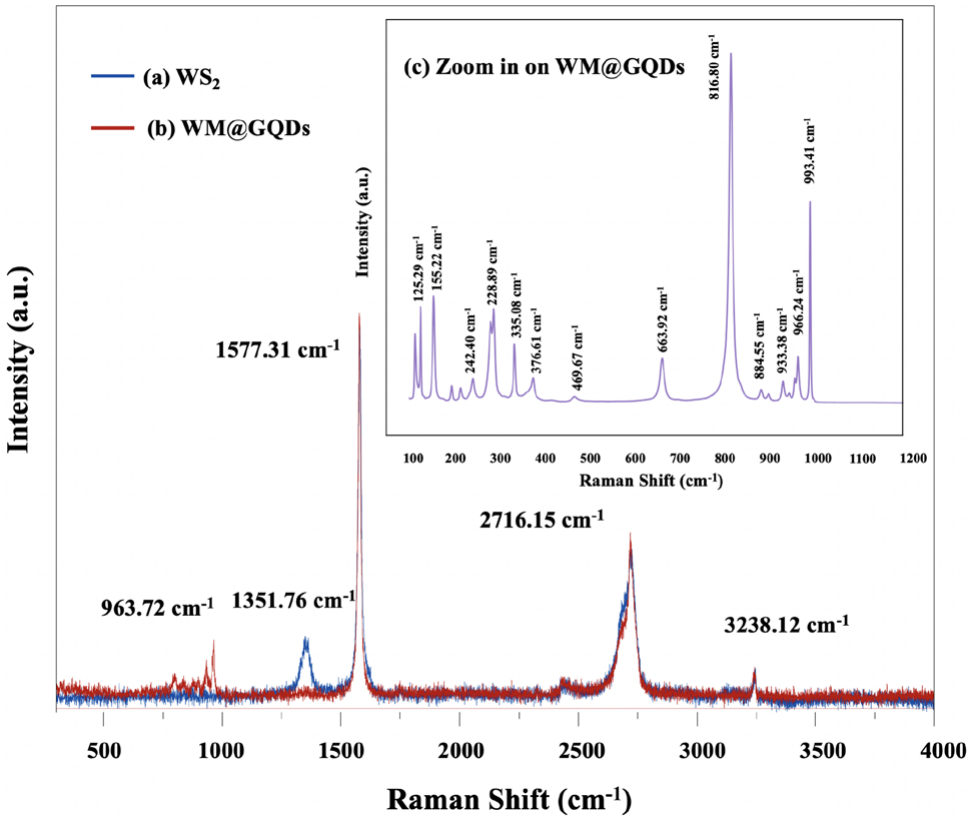
Raman profiles recorded for (**a**) WS_2_, and (**b**) WM@GQD.

The Raman spectra of WS_2_ and WM@GQDs are presented in Fig. 5a,b to study the structure of materials and their interaction property. Figure 5a (blue line) represents the single-domain WS_2_ monolayer and presents three main peaks at 1351. 76, 1577.31, 2716.15, and 32,381.12 cm^−1^. The results were consistent with the reported Raman active modes of WS_2_. The three peaks corresponded to the two second-order longitudinal acoustic (LA) Raman modes at point M, E′2 g, and A1 g of WS_2_, respectively^25^. The peak at 963.72 cm^−1^ represented the first-order peak of E12 g as shown in Fig. 5b. In addition, the zoomed-in peaks of WM@GQDs are shown in the inset figure to confirm the composite structure (Fig. 5c). Two strong peaks were present in the spectral profiles recorded for GQDs (blue line). The G band at 1601 cm^−1^ indicates an ordered planar structure of the sp^2^ carbon atoms (C–C bond), and the D band at 1360 cm^−1^ corresponds to the sp^3^ structural defects (e.g., edges and functional groups). The band ratio of the peak intensity of D and G bands (I_D_/I_G_ = 1.07) can reflect the participation of functional groups at the edges of the GQDs in relation to the C=C bonds in their structures.

#### XPS analysis

**Figure 6:**
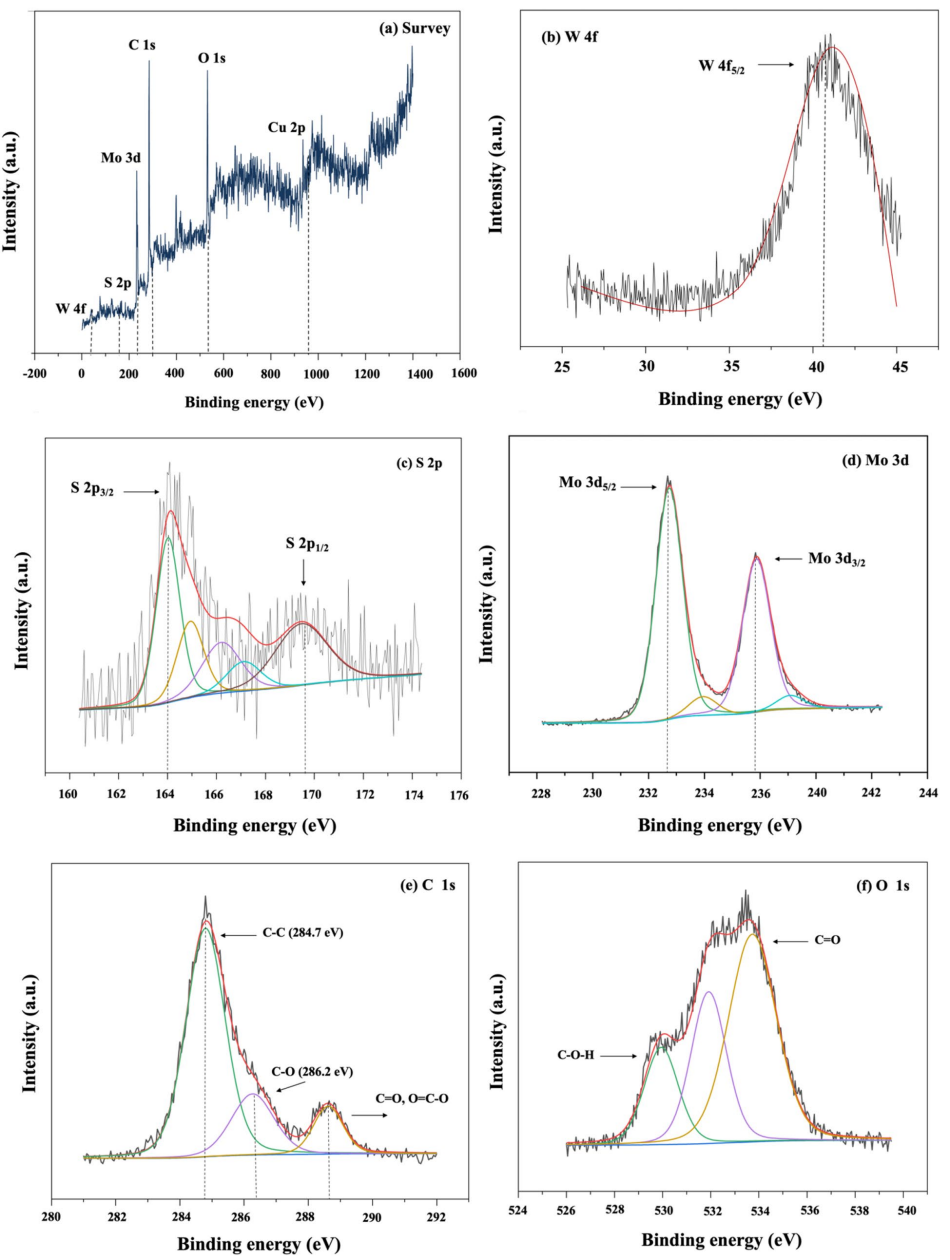
XPS profiles: (**a**) Survey, (**b**) W 4f, (**c**) S 2p, (**d**) Mo 3d, (**e**) C 1s, and (**f**) O 1s profiles.

The elemental compositions, valence states, and oxidation states of the elements in the WM@GQDs were confirmed using the X-ray photoelectron spectroscopy (XPS) technique, as shown in Fig. 6a–e. The XPS survey spectra of the WM@GQDs composite show the presence of W 4f, S 2p, Mo 3d, Cu 2p, C 1s, and O 1s in the hybrid form. There are no additional peaks in the survey spectrum, indicating the absence of impurities in the hybrid. The high-resolution spectrum of W 4f (Fig. 6b) displays a peak with a binding energy of approximately 40.8 eV, which confirms the presence of WS_2_ crystals. The presence of the characteristic 2H phase (with no observable contribution from WOx) was confirmed. The S 2p profiles (Fig. 6c) exhibit the S 2p_3/2_:2p_1/2_ spin–orbit doublet at 164.3 and 169.1 eV, respectively, corresponding to the S^2−^ oxidation state expected for crystalline WS_2_^26^. In addition, Fig. 6d presents the two main peaks in the Mo 3d spectrum at 232.8 and 235.7 eV, which are assigned to Mo 3d_5/2_ and Mo 3d_3/2_, respectively. The C 1s spectrum (Fig. 6e) presents three peaks, corresponding to C–C/C=C at 284.8 eV, C–O at 287.1 eV, and C=O at 289.3 eV^27^. The O 1s spectrum (Fig. 6f) is divided into three sub-bands corresponding to C–O, C=O, and –OH in the GQDs. The XPS analysis confirms the presence of bonded elements present in the WM@GQD composite.

#### Surface area analysis

**Figure 7:**
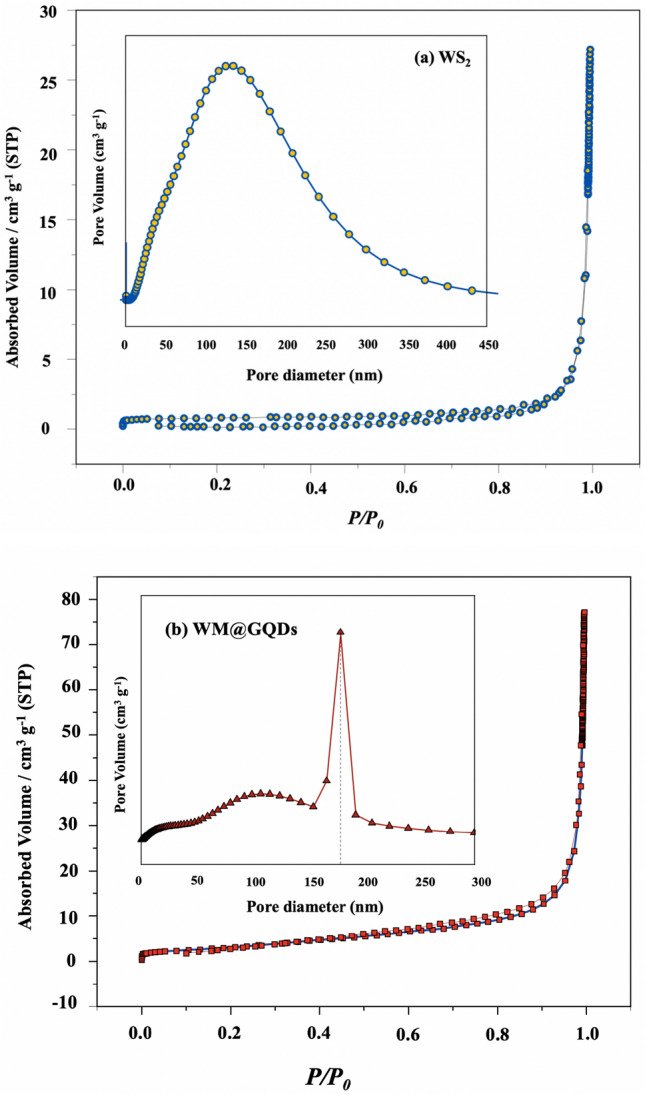
N_2_ adsorption–desorption isotherms recorded for (**a**) WS_2_, and (**b**) WM@GQDs.

The influence of WS_2_ and the WM@GQD composite was studied by analyzing the N_2_ adsorption–desorption isotherms. Figure 7a,b shows that type IV isotherm adsorption curves with H3-type hysteresis loops (according to the IUPAC classification) were recorded for all the samples, indicating that mesoporous structures were formed in all samples^28^. WM@GQDs were characterized by the maximum specific surface area of approximately 69.40 m^2^/g, which was larger than that of WS_2_ (25.30 m^2^/g). The pore diameter of all the samples was primarily distributed within the range of 100–200 nm, as shown in the pore size distribution curves in the inset of Fig. 7. According to the BET results, incorporating GQDs into the WM@GQDs can increase the number of active sites, which can improve the power conversion efficiency of the CEs.

#### Bandgap estimation

**Figure 8:**
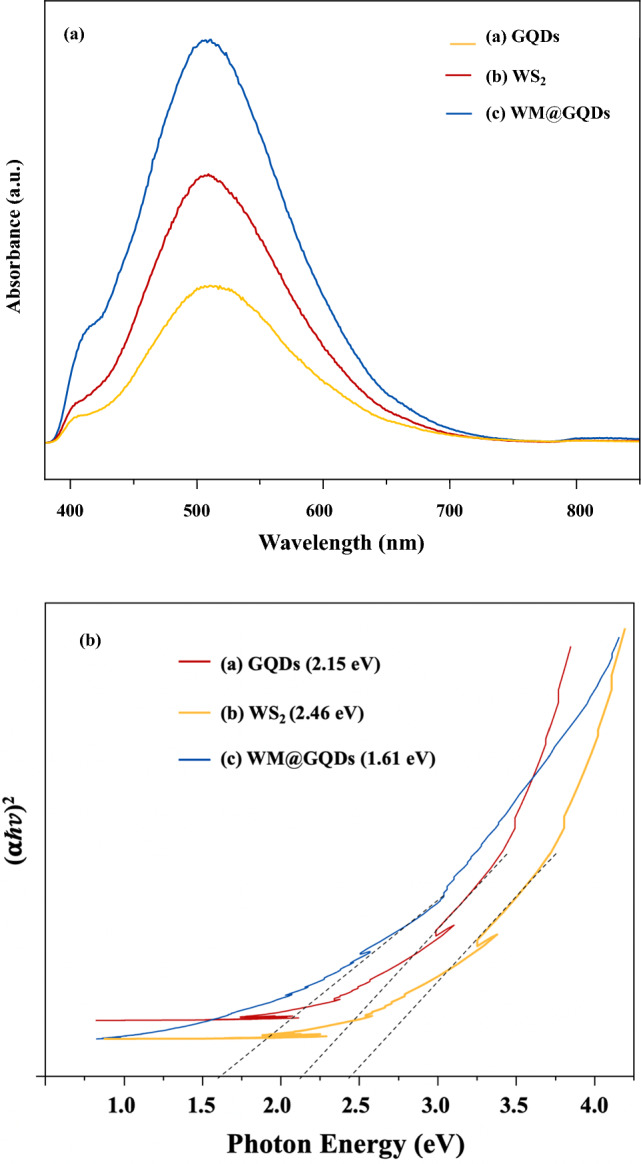
(**a**) UV–Vis profiles and (**b**) Bandgap energy of GQDs, WS_2_, and WM@GQDs.

The UV–Vis spectra of the GQDs, WS_2_, and WM@GQDs are presented in Fig. 8a. The absorption region of a semiconductor is related to its band structure. The band gap energy (Eg) can be obtained from equation^29^.

$${\text{Eg }} = { 124}0/{\lambda} \quad (\lambda\,{\text{is}}\,{\text{incident}}\,{\text{wavelength}}).$$

As shown in Fig. 8a,b, All of them show the best optical absorption performance in the visible light range, and their absorption edge is at approximately 546 nm. corresponding to the bandgap energy (Eg) of 2.46 eV, 2.15 eV, and 1.61 eV for WS_2_, GQDs, and WM@GQDs respectively. The results of UV–Vis DRS confirm that the prepared WM@GQDs composites all have good light absorption properties in the visible light region, which indicates that the visible-light-induced catalytic activity might be effectively enhanced.

### Electrochemical analysis

#### Photovoltaic properties

**Figure 9:**
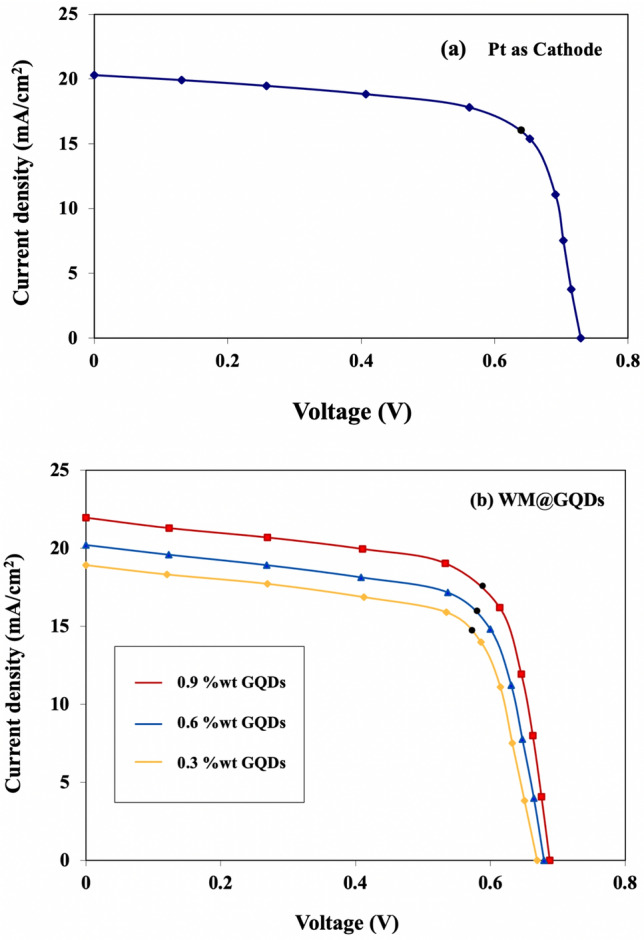
I–V analysis for (**a**) Pt and (**b**) (0.3–0.9%wt) of WM@GQDs.

Electrochemical tests in Fig. 9a,b show the electrochemical activity of (0.3–0.9% wt) WM@GQDs comparable with traditional Pt CE, and the detailed photovoltaic parameters such as open circuit voltage (*Voc*), Fill Factor (FF) and Short circuit current (*Jsc*) are summarized in Table 1. All J–V measurements were carried out under AM 1.5G simulated sunlight using a class AAA solar simulator. From the J–V curves, 0.9%wt WM@GQDs depicts the highest power conversion efficiency (PCE) up to 10.38% (*Voc* = 0.68 V, *Jsc* = 21.97 mA/cm^2^, FF = 0.68) followed by 0.6%wt WM@GQDs which reached power conversion efficiency up to 9.28% (*Voc* = 0.67 V, *Jsc* = 20.21 mA/cm^2^, FF = 0.67), while 0.3%wt WM@GQDs showed a power conversion efficiency of 8.45% (*Voc* = 0.66 V, *Jsc* = 18.93 mA/cm^2^, FF = 0.66) comparable with Pt electrode (10.26%). The higher open circuit voltage is critical for maintaining a higher driving force for electrons around the circuit so as to limit electron–hole recombinations. Moreover, the improvement of power conversion efficiency is due to the larger surface area of the WM@GQDs for I_3_^−^ reduction^30^.

**Table 1:** Photovoltaic parameters of DSSCs with different CEs.

Sample	Voc (V)	Jsc (mA/cm^2^)	FF	PCE (%)	Rs (Ω)	Rct (Ω)
Pt electrode	0.72	20.30	0.69	10.26	12.26	50.68
0.3%wt of WM@GQDs	0.66	18.93	0.66	8.45	12.54	176.21
0.6%wt of WM@GQDs	0.67	20.21	0.67	9.28	12.11	175.44
0.9%wt of WM@GQDs	0.68	21.97	0.68	10.38	12.01	35.24

#### Electrocatalytic and impedance measurements analysis

**Figure 10:**
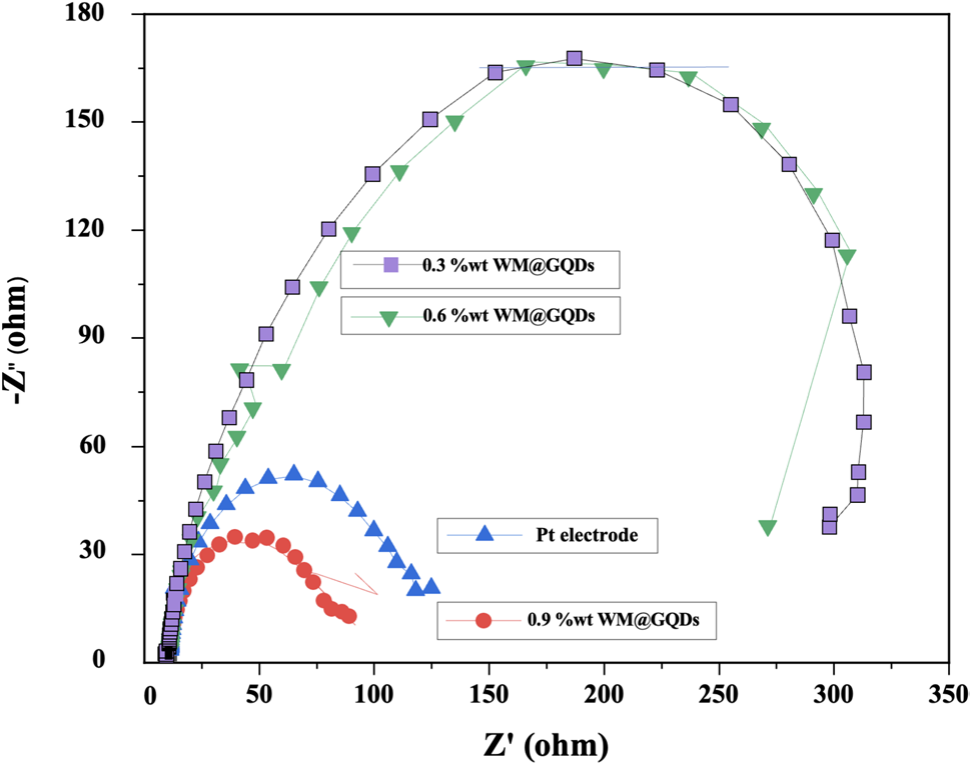
EIS profiles for Pt and (0.3–0.9%wt) of WM@GQDs.

The charge transfer resistance at the counter electrode–electrolyte interface has been investigated through the EIS technique. Figure 10 and Table 1 represent the Nyquist curve for the corresponding (0.3–0.9%wt) WM@GQDs and Pt electrodes with an applied frequency range of 100 MHz and 100 kHz. There are two semi-circles were obtained, the first semicircle represents the charge transfer from FTO to CEs materials and the second semicircle indicates the charge transfer of electrode and electrolyte or electrolyte resistance. From EIS spectra, the charge transfer resistance of 0.9%wt WM@GQDs (35.24 Ω) is lower when compared with 0.3%wt WM@GQDs and 0.6%wt WM@GQDs, which is due to the more electrical conductivity of graphene and high catalytic activity.

#### Mechanism of action of WM@GQDs

**Figure 11:**
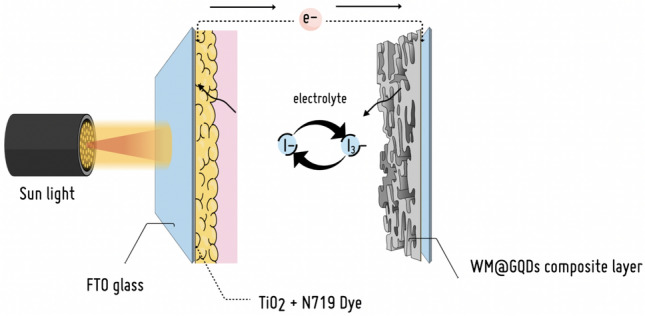
Schematic representation for photovoltaic conversion mechanism of WM@GQDs.

The mechanism of action of the WM@GQDs in the DSSC cell is proposed in Fig. 11. In the first step, when light falls on the photoanode side (TiO_2_ absorbs N719 dyes), the photosensitive dye molecules absorb energy from sunlight. This causes the free electrons in the dye molecules to become excited and jump from the highest occupied molecular orbital (HOMO) to the lowest unoccupied molecular orbital (LUMO). The excited electrons are then injected into the conducting layer, a conduction band of titanium dioxide, and passed on to a layer of translucent conductive glass. Numerous electrons are present in this layer, and when it is connected to an external load, an electric current is generated from the flow of electrons within the cell as they flow through the load to the cathode. This flow of electrons occurs continuously until the cycle is complete^31–33^.

## Conclusion

A novel hybrid composite material, made of tungsten disulfide-molybdenum copper oxide supported with graphene quantum dots (WM@GQDs), was synthesized using a simple and low-cost ultrasonication method. Techniques such as X-ray diffraction analysis, field-emission scanning electron microscopy, and BET analysis were used to confirm the crystallinity and morphology of the material. When used as the 0.9% wt WM@GQDs cathode in a dye-sensitized solar cell, the material had a maximum power conversion efficiency (PCE) of 10.38%, which is higher than that of a Pt electrode (10.26%). Electrochemical impedance spectroscopy showed that the WM@GQDs material had a lower resistance compared to other cathode materials. The unique structure of the hybrid composite, with the interconnection of WS_2_-MoCuO_3_ and GQDs providing a high surface area for maximal dye absorption on the electrode surface, may contribute to its high PCE.

## Supplementary Information

Supplementary Information.

## Data Availability

The datasets used and/or analysed during the current study available from the corresponding author on reasonable request.

## References

[1] Zhao Y, Xu M, Huang X, Lebeau J, Li T, Wang D, Fu H, Fu K, Wang X, Lin J, Jiang H (2022). Toward high efficiency at high temperatures: Recent progress and prospects on InGaN-based solar cells. Mater. Today Energy.

[2] Oh H-J, Dao V-D, Ryu K-H, Lee J-H, Choi H-S (2018). FeSn alloy/graphene as an electrocatalyst for the counter electrode of highly efficient liquid-junction photovoltaic devices. J. Alloys Compd..

[3] Bae K-H, Dao V-D, Choi H-S (2017). Utility of Pt in PtNi alloy counter electrodes as a new avenue for cost effective and highly efficient liquid junction photovoltaic devices. J. Colloid Interface Sci..

[4] Gomathi M, Sankar A, Kannan S, Shkir M, Reddy VRM (2022). Tin selenide/carbon black nanocomposite-based high efficiency counter electrode for dye-sensitized solar cells (DSSCs). Chem. Phys. Lett..

[5] Das A, Nair RG (2021). Fabrication of In_2_O_3_ functionalized ZnO based nanoheterojunction photoanode for improved DSSC performance through effective interfacial charge carrier separation. Opt. Mater..

[6] Dao V-D (2020). Bimetallic PtSe nanoparticles incorporating with reduced graphene oxide as efficient and durable electrode materials for liquid-junction photovoltaic devices. Mater. Today Energy.

[7] Dao V-D, Dang H-LT, Vu NH, Vu HHT, Hoa ND, Hieu NV, Tuan PA (2020). Nanoporous NiO nanosheets-based nanohybrid catalyst for efficient reduction of triiodide ions. Sol. Energy.

[8] Wu M, Lin X, Wang T, Qiu J, Ma T (2011). Low-cost dye-sensitized solar cell based on nine kinds of carbon counter electrodes. Energy Environ. Sci..

[9] Zhou J, Yu X, Jin X, Tang G, Zhang W, Hu J, Zhong C (2014). Novel carbazole-based main chain polymeric metal complexes containing complexes of phenanthroline with Zn(II) or Cd(II): Synthesis, characterization and photovoltaic application in DSSCs. J. Mol. Struct..

[10] Francis MK, Bhargav B, Santhosh N, Govindaraj R, Ahmed N, Balaji C (2021). Bifacial DSSC fabricated using low-temperature processed 3D flower like MoS_2_—High conducting carbon composite counter electrodes. Mater. Today Commun..

[11] Jin L, Wang Y, Wu J, Su C, Zhou H, Xu H (2022). Properties of oxidation quantum dots-CdO/TiO_2_ heterostructures constructed as DSSC photoanodes. Mater. Sci. Semicond. Process..

[12] Wu M, Wang Y, Lin X, Yu N, Wang L, Wang L, Hagfeldtc A, Ma T (2011). Economical and effective sulfide catalysts for dye-sensitized solar cells as counter electrodes. Phys. Chem. Chem. Phys..

[13] Kurniawan D, Weng RJ, Chen YY, Rahardja MR, Nanaricka ZC, Chiang WH (2022). Recent advances in the graphene quantum dot-based biological and environmental sensors. Sens. Actuators.

[14] Chen L, Guo CX, Zhang Q, Lei Y, Xie J, Ee S, Guai G, Song Q, Li CM (2013). Graphene quantum-dot-doped polypyrrole counter electrode for high-performance dye-sensitized solar cells. ACS Appl. Mater. Interfaces.

[15] Wang Z, Zhao C, Gui R, Jin H, Xia J, Zhang F, Xia Y (2016). Synthetic methods and potential applications of transition metal dichalcogenide/graphene nanocomposites. Coord. Chem. Rev..

[16] Balis N, Stratakis E, Kymakis E (2016). Graphene and transition metal dichalcogenide nanosheets as charge transport layers for solution-processed solar cells. Mater. Today.

[17] Yuan X, Zhou B, Zhang X, Li Y, Liu L (2018). Hierarchical MoSe_2_ nanoflowers used as highly efficient electrode for dye-sensitized solar cells. Electrochim. Acta.

[18] Silambarasan K, Harish S, Hara K, Archana J, Navaneethan M (2021). Ultrathin layered MoS_2_ and N-doped graphene quantum dots (N-GQDs) anchored reduced graphene oxide (rGO) nanocomposite-based counter electrode for dye-sensitized solar cells. Carbon.

[19] Ebrahimi M, Soleimanian V, Ghasemi M, Nekoeinia M, Mokhtari A (2023). Effects of graphene quantum dots on microstructure, optical and gas sensing properties of coral-like ZnCo_2_O_4_ nanoparticles. Phys. B Condens. Matter..

[20] Rajamanickam N, Soundarrajan P, Senthil Kumar SM, Jayakumar K, Ramachandran K (2019). Boosting photo charge carrier transport properties of perovskite BaSnO_3_ photoanodes by Sr doping for enhanced DSSCs performance. Electrochim. Acta..

[21] Nie Y, Bao R, Yi J, Tao J, Liu P, Ma R, Luo H, Ma D (2022). Highly efficient heterostructures of C3N4 and o-GQDs with enrichment of specific oxygen-containing groups for photocatalytic applications. J. Alloys Compd..

[22] Grützmacher PG, Schranz M, Hsu CJ, Bernardi J, Steiger-Thirsfeld A, Hensgen L, Ripoll MR, Gachot C (2022). Solid lubricity of WS_2_ and Bi_2_S_3_ coatings deposited by plasma spraying and air spraying. Surf. Coat. Technol..

[23] Durai L, Kong CY, Badhulika S (2020). One-step solvothermal synthesis of nanoflake-nanorod WS_2_ hybrid for non-enzymatic detection of uric acid and quercetin in blood serum. Mater. Sci. Eng. C.

[24] Fatima T, Husain S, Narang J, Khanuja Shetti M, Raghava Reddy NPK (2022). Novel tungsten disulfide (WS_2_) nanosheets for photocatalytic degradation and electrochemical detection of pharmaceutical pollutants. J. Water Process. Eng..

[25] Barbosa AN, Figueroa NS, Giarola M, Mariotto G, Freire FL (2020). Straightforward identification of monolayer WS_2_ structures by Raman spectroscopy. Mater. Chem. Phys..

[26] Omelianovych O, Dao V-D, Larina LL, Choi H-S (2016). Optimization of the PtFe alloy structure for application as an efficient counter electrode for dye-sensitized solar cells. Electrochim. Acta.

[27] Zhang H, Liu T, Huang Z, Cheng J, Wang H, Zhang D, Ba X, Zheng G, Yan M, Cao M (2022). Engineering flexible and green electromagnetic interference shielding materials with high performance through modulating WS_2_ nanosheets on carbon fibers. J. Materiomics..

[28] Li Y, Zhou Y, Wang Y, Liu M, Yuan J, Men X (2021). Facile synthesis of WS_2_@GO nanohybrids for significant improvement in mechanical and tribological performance of EP composites. Tribol. Int..

[29] Zhong Y, Shao Y, Ma F, Wu Y, Huang B, Hao X (2017). Band-gap-matched CdSe QD/WS_2_ nanosheet composite: Size-controlled photocatalyst for high-efficiency water splitting. Nano Energy.

[30] Dao V-D, Kim S-H, Choi H-S, Kim J-H, Park H-O, Lee J-K (2011). Efficiency enhancement of dye-sensitized solar cell using Pt hollow sphere counter electrode. J. Phys. Chem. C.

[31] Wu M, Lin Y, Guo H, Li W, Wang Y, Lin X (2015). Design a novel kind of open-ended carbon sphere for a highly effective counter electrode catalyst in dye-sensitized solar cells. Nano Energy.

[32] Wu M, Sun M, Zhou H, Ma J-Y, Ma T (2020). Carbon counter electrodes in dye-sensitized and perovskite solar cells. Adv. Funct. Mater..

[33] Hora C, Santos F, Pereira AMVM, Sales MGF, Ivanou D, Mendes A (2022). PEDOT-graphene counter-electrode for solar and improved artificial light conversion in regular, bifacial and FTO-less cobalt mediated DSSCs. Electrochim. Acta.

