# Supplementary material for: A novel of WS2–MoCuO3 supported with graphene quantum dot as counter electrode for dye-sensitized solar cells application
Source: Sci Rep. 2023 May 12;13:7762. doi: 10.1038/s41598-023-34637-3 (PMC10182016; doi:10.1038/s41598-023-34637-3)
Supplement: Supplementary file 1 — Supplementary Information. [file 41598_2023_34637_MOESM1_ESM.docx]

**Supporting Information**

**1. Stability Analysis**

The stability of the counter electrode in a DSSC can be measured using various electrochemical and physicochemical techniques. In this paper, some of the commonly used methods are Cyclic voltammetry (CV). The results show that 40 successive cyclic voltammetry tests were conducted to determine its stability in the electrolyte and find out if there is a reduction in peak reduction current density over the 40 cycles relative to the platinum electrode as indicated by a limited reduction in the value of the peak reduction current density. Moreover, there is no change in the shape of the CV curve over the range of the stability test indicative of sustained stability in the electrolyte whilst there is fluctuation in

reduction current density for the platinum electrode.


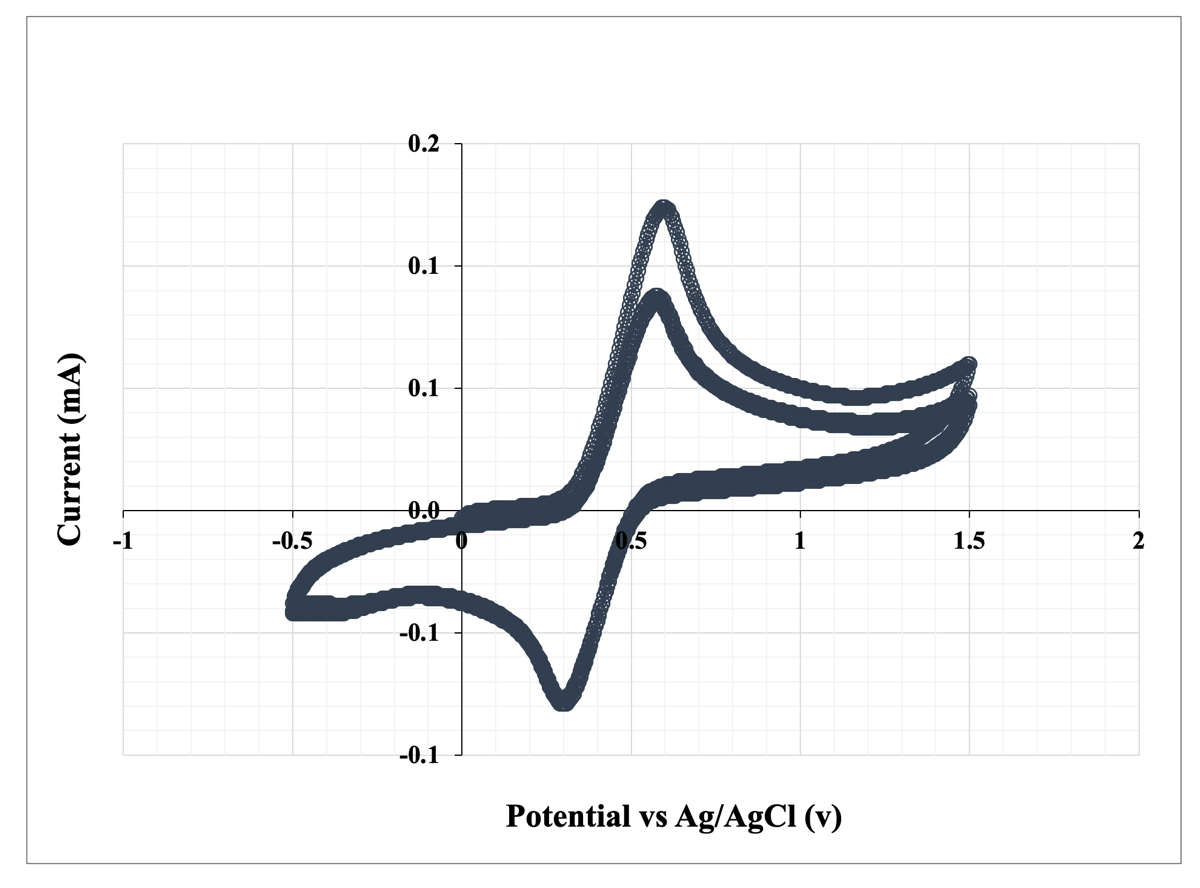


Cyclic voltammetry (CV) analysis curves of .9 %wt MW@GQDs for 40 cycles
